# Characteristics of four commonly used self-expanding biliary stents: an *in vitro* study

**DOI:** 10.1186/s41747-024-00425-5

**Published:** 2024-02-19

**Authors:** Jiaywei Tsauo, Yan Fu, Yue Liu, Xiaowu Zhang, He Zhao, Xiao Li

**Affiliations:** 1 Department of Interventional Therapy, National Cancer Center/National Clinical Research Center for Cancer/Cancer Hospital, Chinese Academy of Medical Sciences and Peking Union Medical College, Beijing, 100021 China; 2 Department of Interventional Radiology, Guangdong Provincial People’s Hospital (Guangdong Academy of Medical Sciences), Southern Medical University, Guangzhou, Guangdong 510080 China; 3 Department of Etiology and Carcinogenesis and State Key Laboratory of Molecular Oncology, National Cancer Center/National Clinical Research Center for Cancer/Cancer Hospital, Chinese Academy of Medical Sciences and Peking Union Medical College, Beijing, 100021 China

**Keywords:** Biliary tract, Digestive system diseases, Jaundice (obstructive), Prostheses and implants, Self-expandable metallic stents

## Abstract

**Background:**

Knowledge of the characteristics of self-expanding metal stents (SEMSs) is essential during selection process to ensure the best therapeutic outcomes for patients with malignant biliary obstruction. The aim of this study was to evaluate the characteristics of four commonly used SEMSs.

**Methods:**

This *in vitro* study analyzed the radial force (RF), crush resistance (CR), axial force (AF), conformability, surface quality, foreshortening, and radiopacity of the following SEMSs: uncovered Wallflex™, EGIS single bare, Zilver 635®, and E-Luminexx™. Two samples of each SEMS type were included in this study, all having identical specifications with a diameter of 10 mm and a length of 6 cm. One sample from each type was analyzed for surface quality, followed by CR, conformability, and foreshortening. The other sample was analyzed for radiopacity, followed by RF and AF.

**Results:**

The uncovered Wallflex™ exhibited low RF, high CR, high AF, good conformability, poor surface quality, high foreshortening, and good radiopacity. The EGIS single bare demonstrated high RF, high CR, low AF, moderate conformability, good surface quality, high foreshortening, and poor radiopacity. The Zilver 635® displayed moderate RF, low CR, low AF, moderate conformability, moderate surface quality, no foreshortening, and good radiopacity. The E-Luminexx™ showed high RF, moderate CR, high AF, poor conformability, poor surface quality, no foreshortening, and good radiopacity.

**Conclusions:**

There was considerable variation in the characteristics among the four evaluated SEMSs. These characteristics should be carefully considered during selection to ensure optimal therapeutic outcomes for patients.

**Relevance statement:**

The selection of self-expanding metal stents for treating malignant biliary obstruction requires careful consideration of various characteristics, including their radial force, crush resistance, axial force, conformability, surface quality, foreshortening, and radiopacity.

**Key points:**

• The characteristics of self-expanding metal stents (SEMSs) can vary considerably.

• Specific situations may warrant the use of SEMSs with particular characteristics over others.

• Characteristics of SEMSs must be considered during selection for optimal outcomes.

**Graphical Abstract:**

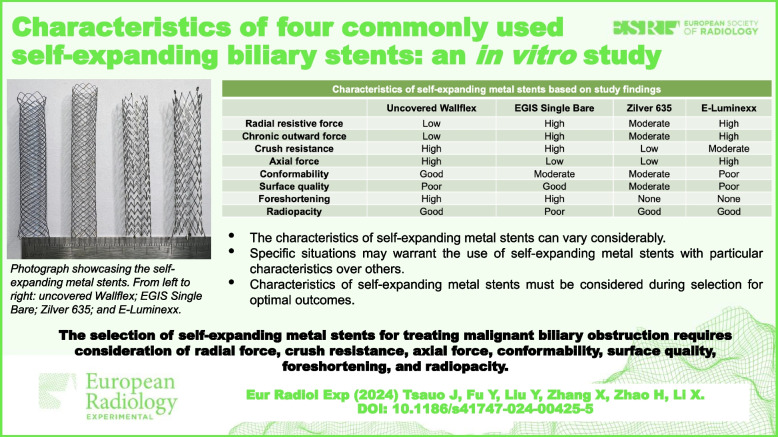

## Background

Self-expanding metal stents (SEMSs) are widely used for the treatment of malignant biliary obstruction [1, 2]. Over the years, numerous studies have been conducted to compare the outcomes of various types of SEMSs for this application [3–10]. However, despite the considerable number of studies available, a consensus regarding the optimal choice of SEMS has not yet been reached [1, 2]. Nonetheless, it is well known that the characteristics of SEMSs can vary considerably due to differences in design (*e.g.,* closed-cell or open-cell configuration) and manufacturing processes (*e.g.,* braiding, laser cutting, or knitting) [11, 12]. Furthermore, specific situations (*e.g.,* intrinsic or extrinsic strictures) may warrant the use of SEMSs with particular characteristics over others.

Key characteristics that merit careful consideration by physicians include radial force (RF), crush resistance (CR), axial force (AF), conformability, surface quality, foreshortening, and radiopacity [13]. However, data pertaining to this information are often not publicly available. This situation presents a challenge for us physicians as it hinders our ability to make well-informed decisions when selecting SEMS for our patients.

To address this issue, we conducted an *in vitro* study to evaluate the characteristics of four commonly used SEMSs in the treatment of malignant biliary obstruction.

## Methods

This study did not require institutional review board approval.

### Self-expanding metal stent selection and analyzed characteristics

**Fig. 1:**
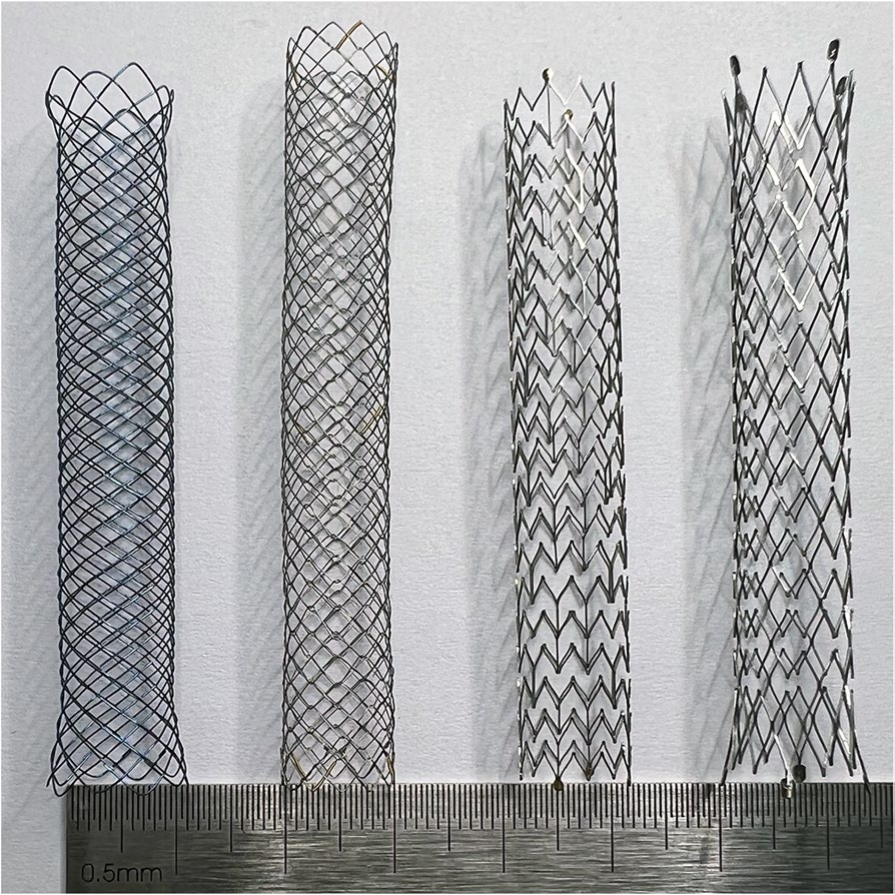
Photograph showcasing the self-expanding metal stents. From left to right: uncovered Wallflex™ Biliary RX Stent, EGIS Single Bare Biliary Stent, Zilver 635® Biliary Self-Expanding Stent, and E-Luminexx™ Biliary Stent

In this study, we have chosen to analyze the following four SEMSs: uncovered Wallflex™ Biliary RX Stent (Boston Scientific, Natick, MA, USA), EGIS Single Bare Biliary Stent (S&G Biotech, Yongin, Gyeonggi-do, Korea), Zilver 635® Biliary Self-Expanding Stent (Cook Medical, Bloomington, IN, USA), and E-Luminexx™ Biliary Stent (BD, Tempe, Arizona, USA) (Fig. 1). These SEMSs were selected because they are four of the most used in our country for the treatment of malignant biliary obstruction. Covered SEMSs were not selected due to their infrequent usage in our country. This can be attributed to a limited body of research demonstrating clear advantages of covered SEMSs over uncovered SEMSs [14–18]. To ensure uniformity in the analysis, all the SEMSs used had identical specifications, with a diameter of 10 mm and a length of 6 cm. Table 1 presents the design and manufacturing information for these SEMSs.

**Table 1:** Design and manufacturing information of the self-expanding metal stents

Name	Material	Type	Struct thickness	Anti-migration features	Radiopaque markers	Weight	Delivery system	Reconstrainable
Uncovered Wallflex™	Platinum-cored nitinol wire	Braided, closed cell	180 μm	Flared ends	None	0.46 g	8 Fr	Up to 80% deployment
EGIS Single Bare	Nitinol wire	Knitted, closed cell	125 μm	None	Both ends, middle point	0.23 g	8 Fr	Up to 80% deployment
Zilver 635®	Nitinol tube	Laser cut, open cell	190 μm	None	Both ends	0.23 g	6 Fr	No
E-Luminexx™	Nitinol tube	Laser cut, open cell	220 μm	Flared ends	Both ends	0.32 g	6 Fr	No

One sample of each SEMS type was provided for free by the manufacturers and was analyzed for surface quality, CR, conformability, and foreshortening. Another sample of each type, purchased from local distributors, was analyzed for radiopacity, followed by RF and AF. All analyses were conducted by three authors (J.T., Y.F., and Y.L.) with assistance from laboratory technicians who were familiar with the testing equipment. Statistical analysis was not performed since each analysis included only one sample of each SEMS type. Table 2 summarizes the definitions and clinical importance of the characteristics analyzed in this study.

**Table 2:** Definition and clinical relevance of the stent characteristics analyzed

	**Definition**	**Clinical relevance**
**Radial resistive force**	The ability of a stent to resist radial compression	High: Maintains lumen patency in concentric strictures
**Chronic outward force**	The continuous outwards radial force exerted by a stent	High: Ensures apposition in concentric strictures and reduces stent migration
**Crush resistance**	The ability of a stent to resist unidirectional compression	High: Maintains lumen patency in eccentric strictures
**Axial force**	The reactive force exerted by a stent when it is bent axially	Low: Prevents straightening or kinking in tortuous strictures
**Conformability**	The ability of a stent to maintain lumen patent when it is bent axially	High: Maintains lumen patency in tortuous strictures
**Surface quality**	The physical smoothness of a stent’s surface	Good: Reduces corrosion, bacterial adherence, and biofilm formation
**Foreshortening**	The change in length of a stent as it is compressed radially	None: Facilitates accurate placement of the stent
**Radiopacity**	The extent to which a stent is visible under fluoroscopy	Good: Facilitates accurate placement of the stent

### Radial force analysis

The radial force of the SEMSs was evaluated using an automated RF testing machine (TTR2, Blockwise Engineering LLC, Tempe, AZ, USA). Before placing each SEMS into the crimper of the machine, the electric heaters within the compression dies were set to 37 °C. The machine was then programmed to compress the SEMS from a diameter of 12 mm down to 3 mm before allowing it to return to its uncompressed state. During this process, both the force required for compression, termed the radial resistive force (RRF), and the force exerted by the SEMS during its return to the uncompressed state, known as the chronic outward force (COF), were recorded. This process was carried out three times for each SEMS type.

### Crush resistance analysis

The crush resistance (CR) of each SEMS was evaluated using an automated force testing machine (PT-1171B; Perfect International Instrument, Taipei, Taiwan, China). The SEMS was positioned on the bottom plate of the machine, and the upper plate was programmed to descend toward the SEMS until a 5-mm gap remained between the plates. The force applied during this process was recorded, representing the CR for the SEMS. This assessment was conducted three times for each SEMS type.

### Axial force analysis

The AF of the SEMSs was evaluated using a method consistent with prior research [11, 12]. Each SEMS was firmly secured in a glass tube, with a 40-mm segment remaining flexible. An automated force testing machine (PT-1171B; Perfect International Instrument) was then used to exert a perpendicular force on this flexible segment until a 60° angle was achieved. The force required to maintain the flexible segment at this angle was recorded and signified the AF of the SEMS. This evaluation procedure was performed in triplicate for each SEMS type.

### Conformability analysis

The conformability of the SEMSs was evaluated using three rigid cylindrical tube models, shaped into L, U, and S configurations (constructed in-house). The inner diameter of each model was 10 mm. For the assessment, each SEMS was meticulously positioned within the respective model, and the distances between the stent and the wall at the curves were precisely measured. The entire analysis was performed three times for each SEMS type.

### Surface quality analysis

A scanning electron microscope (TM-1000. Hitachi, Tokyo, Japan) was used to inspect the surface of each SEMS for defects. These defects included, but were not limited to, slag, pitting, pores, cracks, and abrasion tracks. Nine random locations within each stent were inspected, and the surface quality of each SEMS was scored by three authors independently. The scoring was based on a scale defined as follows: 1 = poor quality, 2 = moderate quality, 3 = good quality, and 4 = very good quality.

### Foreshortening analysis

A semitransparent polytetrafluoroethylene tube with an inner diameter of 2.5 mm was used to assess foreshortening. First, the length of each SEMS was accurately measured. Afterward, each SEMS was compressed and carefully loaded into the tube, at which point a precise second measurement of its length was taken. The foreshortening was then calculated using the following equation: percent foreshortening = (change in length ÷ loaded length) × 100. The entire procedure, including compression, loading, and measuring, was performed three times to ensure the accuracy of the foreshortening calculations.

### Radiopacity analysis

The fluoroscopic images of the SEMSs were obtained using an Allura Xper FD20 x-ray system (Philips Healthcare, Best, the Netherlands). A 15-cm-thick polymethyl methacrylate block was positioned above the SEMSs, and the distance between the flat panel detector and the block was adjusted to 100 cm. Fluoroscopic images were then captured in three distinct modes: low dose, normal dose, and high dose, with tube voltages of 75 kVp, 67 kVp, and 66 kVp, respectively. Three authors (J.T., X.Z., and X.L.) independently evaluated the radiopacity of each SEMS using the following scoring system: 0 = not visible; 1 = poor visibility; 2 = moderate visibility; 3 = good visibility; and 4 = very good visibility.

## Results

### Radial force analysis

**Fig. 2:**
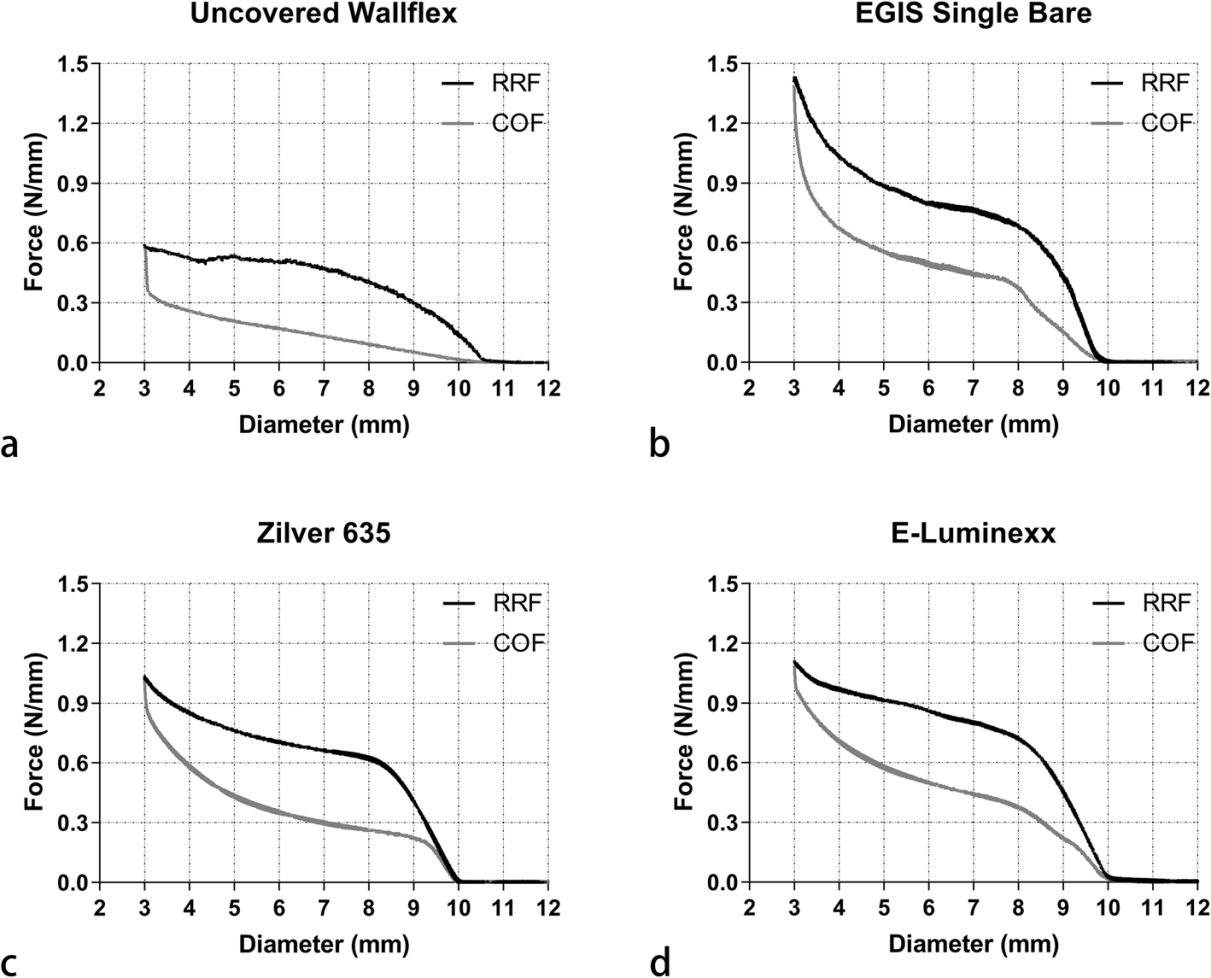
Line graphs illustrating the RRF and COF of uncovered Wallflex™ Biliary RX Stent (**a**), EGIS Single Bare Biliary Stent (**b**), Zilver 635® Biliary Self-Expanding Stent (**c**), and E-Luminexx™ Biliary Stent (**d**) *RRF* Radial resistive force, *COF* Chronic outward force

All SEMSs demonstrated an increase in RRF as their diameters decreased and a decrease in COF as their diameters increased (Fig. 2). However, the rate and pattern of these changes varied among the different SEMSs. The RRF of the uncovered Wallflex™ Biliary RX Stent increased subtly from 0.135 N/mm at 10-mm diameter to 0.586 N/mm at 3-mm diameter. Its COF saw an initial steep decline, followed by a more gradual reduction, moving from 0.578 N/mm at 3-mm diameter to 0.015 N/mm at 10-mm diameter. The EGIS Single Bare Biliary Stent saw a sharp increase in RRF from 0.006 N/mm at 10-mm diameter to 0.680 N/mm at 8-mm diameter. This increase subsequently slowed before experiencing another surge, rising from 0.885 N/mm at 5-mm diameter to 1.409 N/mm at 3-mm diameter. Meanwhile, the COF sharply decreased from 1.39 N/mm at 3-mm diameter to 0.558 N/mm at 5-mm diameter. It then saw another sharp drop from 0.373 N/mm at 8-mm diameter to 0.001 N/mm at 10-mm diameter. For the Zilver 635® Biliary Self-Expanding Stent, the RRF increased sharply from 0.012 N/mm at 10-mm diameter to 0.619 N/mm at 8-mm diameter, subsequently rising more gradually to 1.022 N/mm at 3-mm diameter. Its COF exhibited a more linear decrease, transitioning from 1.014 N/mm at 3-mm diameter to 0.002 N/mm at 10-mm diameter. Similarly, the E-Luminexx™ Biliary Stent experienced a sharp increase in RRF, from 0.029 N/mm at 10-mm diameter to 0.720 N/mm at 8-mm diameter, subsequently increasing more gradually to 1.101 N/mm at 10-mm diameter. Concurrently, its COF had a linear reduction, moving from 1.089 N/mm at 3-mm diameter to 0.223 N/mm at 10-mm diameter.

### Crush resistance analysis

**Fig. 3:**
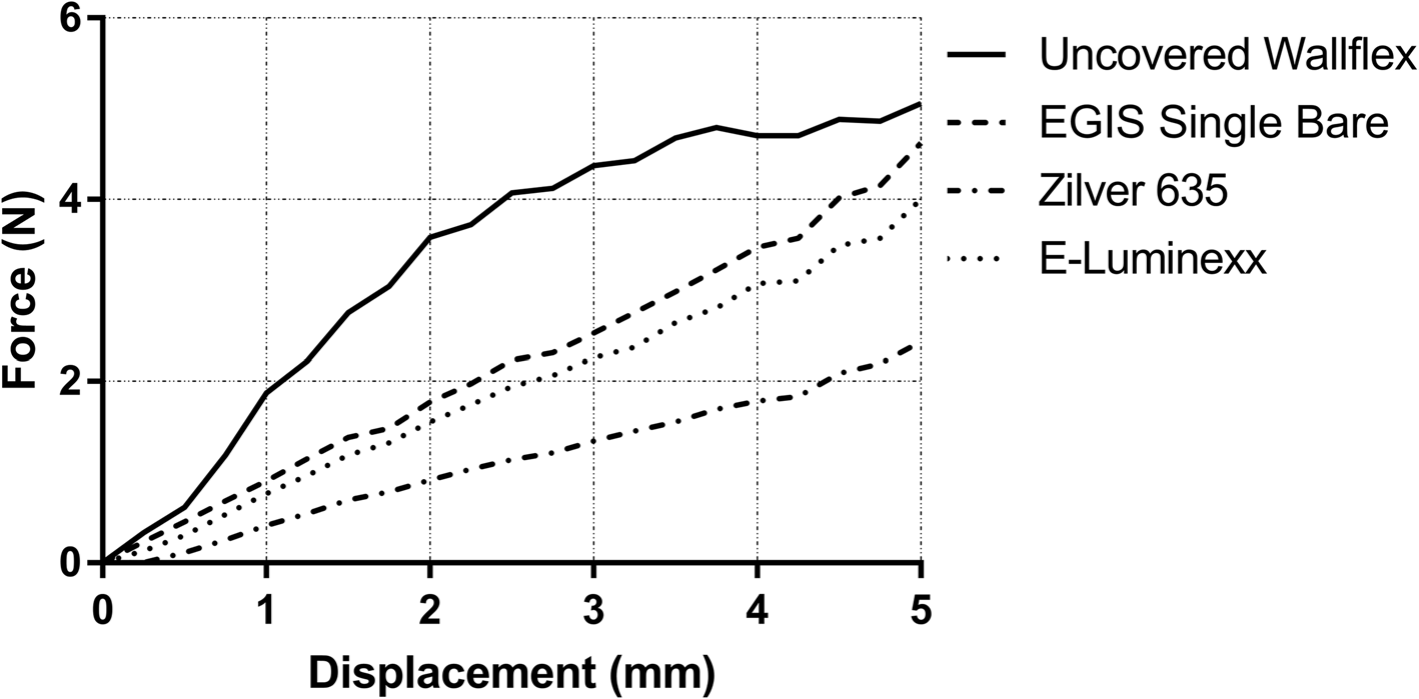
Line graph illustrating the crush resistance of self-expanding metal stents

All SEMSs exhibited an increase in CR upon displacement from 0 to 5 mm (Fig. 3). Among these, the uncovered Wallflex™ Biliary RX Stent recorded the highest CR value. Following closely were the EGIS Single Bare Biliary Stent and the E-Luminexx™ Biliary Stent, while the Zilver 635® Biliary Self-Expanding Stent recorded the lowest CR value. Notably, the CR value of the uncovered Wallflex™ Biliary RX Stent saw a sharp increase to 4.37 ± 0.11 N (mean ± standard deviation) during displacement from 0 to 3 mm. However, after this point, the rate of increase in CR decreased, leading to a CR value of 5.06 ± 0.18 N at displacement of 5 mm. Conversely, the EGIS Single Bare Biliary Stent, the E-Luminexx™ Biliary Stent, and the Zilver 635® Biliary Self-Expanding Stent demonstrated a consistent linear increase in CR values. When displaced from 0 to 5 mm, these SEMSs achieved CR values of 4.62 ± 0.14 N, 4.00 ± 0.18 N, and 2.43 ± 0.05 N, respectively.

### Axial force analysis

**Fig. 4:**
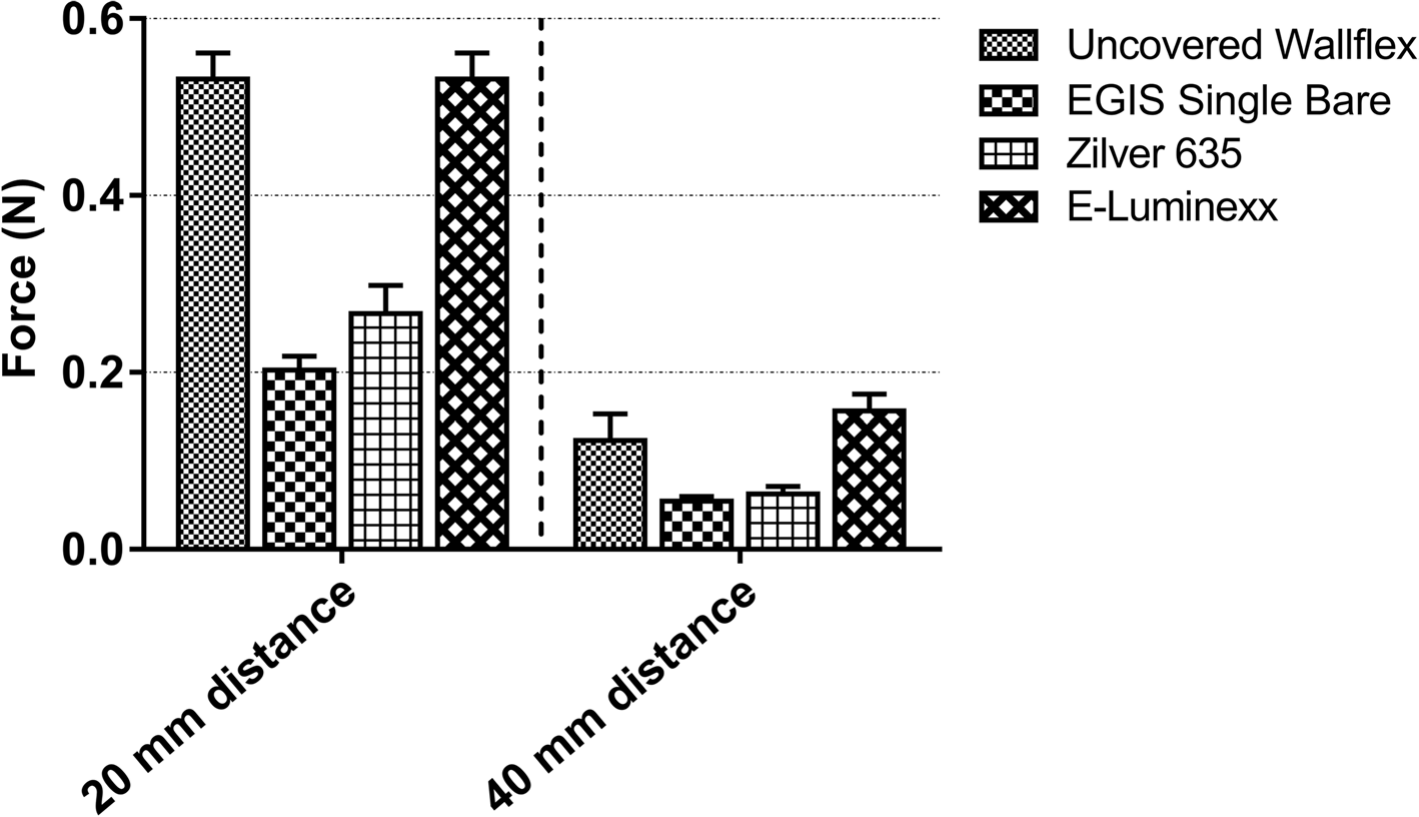
Bar graph illustrating the axial force of self-expanding metal stents. The error bars represent the standard deviation of six measurements

All SEMSs exhibited a considerably higher AF at 20 mm from the bending point compared to 40 mm (Fig. 4). Among these, the EGIS Single Bare Biliary Stent demonstrated the lowest AF values, recording 0.202 ± 0.016 N and 0.055 ± 0.005 N (mean ± standard deviation) at 20-mm and 40-mm distances, respectively. The Zilver 635® Biliary Self-Expanding Stent followed closely, with recorded AF values of 0.266 ± 0.032 N and 0.063 ± 0.009 N at the corresponding distances. On the other hand, the uncovered Wallflex™ Biliary RX Stent and the E-Luminexx™ Biliary Stent recorded the highest AF values. Interestingly, the uncovered Wallflex™ Biliary RX Stent had a slightly higher AF value at the 20-mm distance (0.532 ± 0.029 N) compared to the E-Luminexx™ Biliary Stent (0.490 ± 0.022 N). However, this trend was reversed at the 40-mm distance, with the E-Luminexx™ Biliary Stent displaying a marginally higher AF value (0.156 ± 0.019 N) than the uncovered Wallflex™ Biliary RX Stent (0.123 ± 0.030 N).

### Conformability analysis

**Fig. 5:**
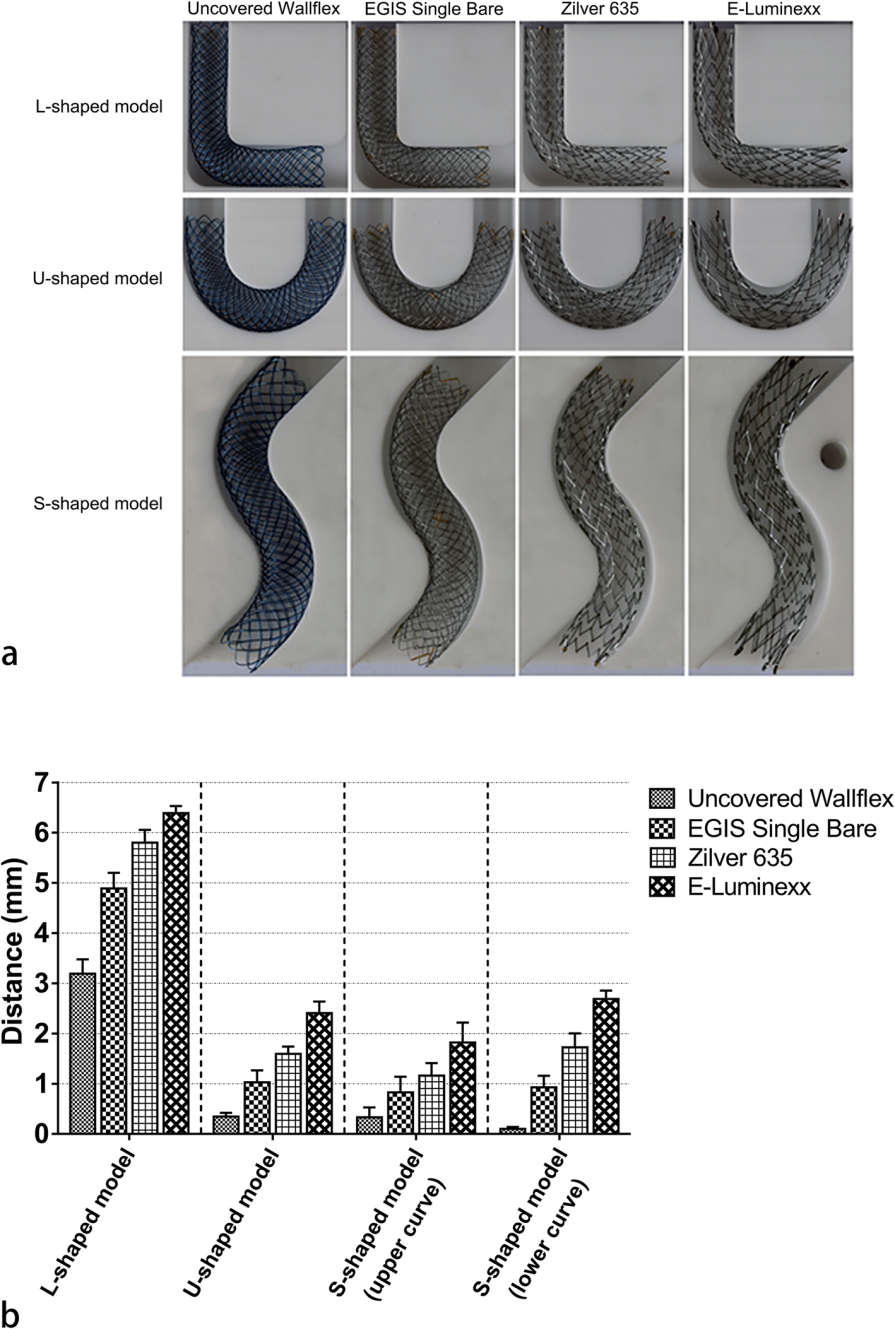
**a** Photographs showcasing the conformability of self-expanding metal stents. **b** Bar graph illustrating the stent-to-wall distances at the curves. The error bars represent the standard deviation of three measurements

In both the L-shaped and U-shaped models, the shortest stent-to-wall distances at the curves were recorded by the uncovered Wallflex™ Biliary RX Stent, with values of 3.193 ± 0.096 mm (mean ± standard deviation) and 0.346 ± 0.072 mm, respectively (Fig. 5). This was followed sequentially by the EGIS Single Bare Biliary Stent (4.891 ± 0.141 mm and 1.029 ± 0.204 mm, respectively), the Zilver 635® Biliary Self-Expanding Stent (5.800 ± 0.257 mm and 1.598 ± 0.049 mm, respectively), and the E-Luminexx™ Biliary Stent (6.390 ± 0.050 mm and 2.409 ± 0.188 mm, respectively). A similar pattern was observed in the S-shaped model, where, again, the uncovered Wallflex™ Biliary RX Stent recorded the smallest stent-to-wall distances at both the upper and lower curves (0.331 ± 0.171 mm and 0.104 ± 0.020 mm, respectively), followed by the EGIS Single Bare Biliary Stent (0.830 ± 0.238 mm and 0.925 ± 0.165 mm, respectively), the Zilver 635® Biliary Self-Expanding Stent (1.160 ± 0.224 mm and 1.725 ± 0.275 mm, respectively), and the E-Luminexx™ Biliary Stent (1.820 ± 0.350 mm and 2.687 ± 0.150 mm, respectively).

### Surface quality analysis

**Fig. 6:**
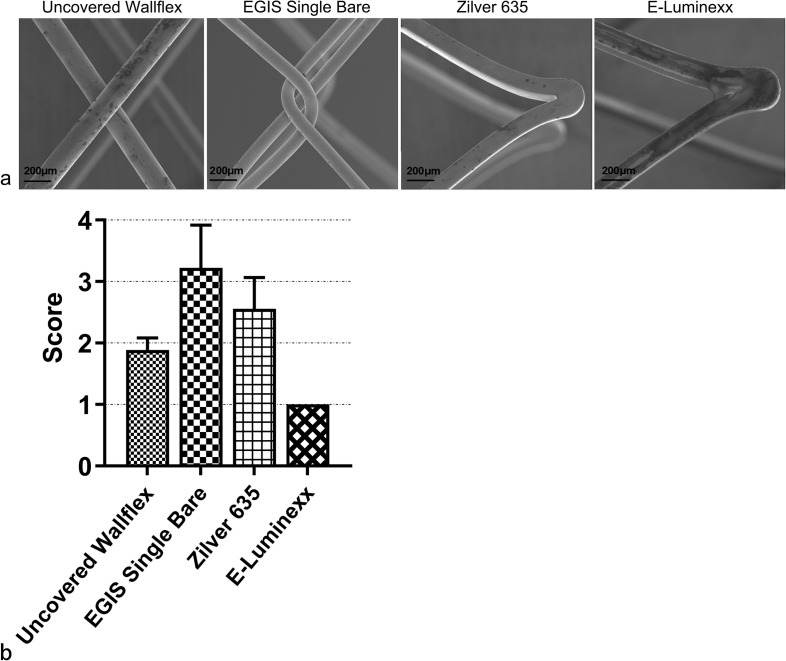
**a** Scanning electron microscope images showcasing the surface quality of self-expanding metal stents. **b** Bar graph illustrating the subjective surface quality scores of self-expanding metal stents. The error bars represent the standard deviation of three assessments

The EGIS Single Bare Biliary Stent achieved a notably high subjective surface quality score of 3.2 ± 0.7 (mean ± standard deviation) (Fig. 6). Following closely behind was the Zilver 635® Biliary Self-Expanding Stent, which obtained a score of 2.6 ± 0.5. In contrast, the uncovered Wallflex™ Biliary RX Stent and the E-Luminexx™ Biliary Stent displayed low subjective surface quality scores of 1.9 ± 0.2 and 1.0 ± 0.0, respectively.

### Foreshortening analysis

**Fig. 7:**
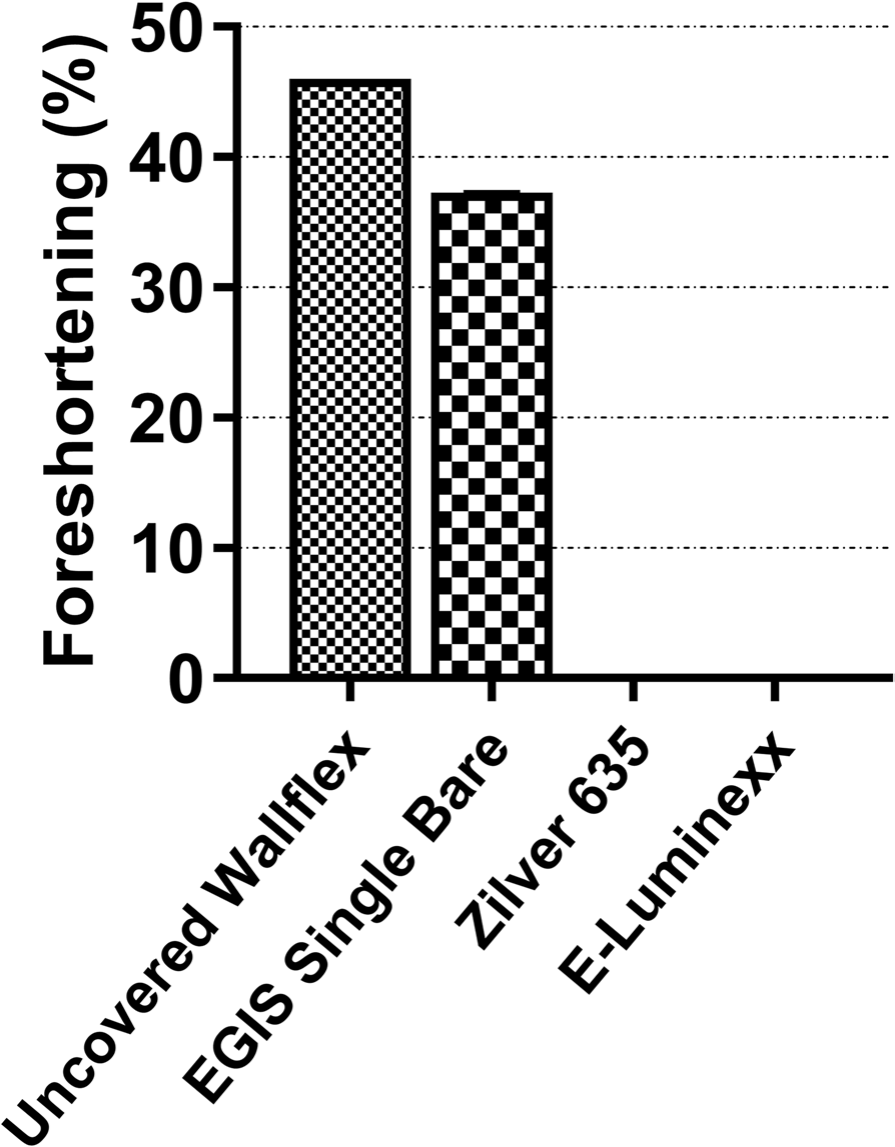
Bar graph illustrating the foreshortening of self-expanding metal stents. The error bars represent the standard deviation of three measurements

The uncovered Wallflex™ Biliary RX Stent displayed a high degree of foreshortening, measuring at 45.7 ± 0.1% (mean ± standard deviation) (Fig. 7). The EGIS Single Bare Biliary Stent closely followed with a foreshortening value of 37.0 ± 0.4%. In contrast, neither the Zilver 635® Biliary Self-Expanding Stent nor the E-Luminexx™ Biliary Stent demonstrated any foreshortening.

### Radiopacity analysis

**Fig. 8:**
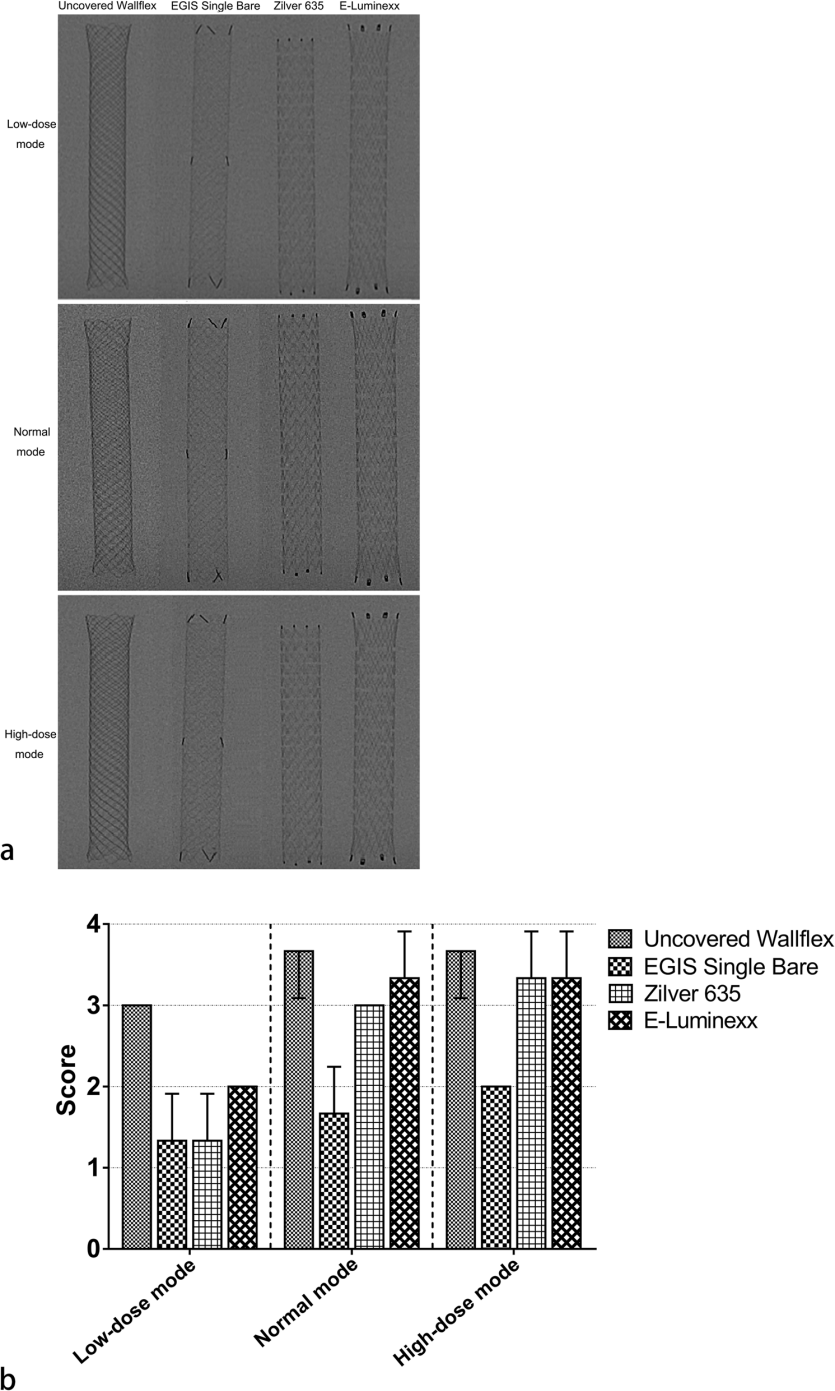
**a** Fluoroscopic images showcasing the radiopacity of self-expanding metal stents. **b** Bar graph illustrating the subjective radiopacity scores of self-expanding metal stents. The error bars represent the standard deviation of three assessments

The uncovered Wallflex™ Biliary RX Stent consistently received high subjective radiopacity scores in low-dose, normal, and high-dose modes (3.0 ± 0.0 [mean ± standard deviation], 3.7 ± 0.6, and 3.7 ± 0.6, respectively) (Fig. 8). On the contrary, the EGIS Single Bare Biliary Stent scored low across all three modes (1.3 ± 0.6, 1.7 ± 0.6, and 2.0 ± 0.0, respectively). Interestingly, the Zilver 635® Biliary Self-Expanding Stent and the E-Luminexx™ Biliary Stent both earned high scores in the normal (3.0 ± 0.0 and 3.3 ± 0.6, respectively) and high-dose modes (3.3 ± 0.6 and 3.3 ± 0.6, respectively). Yet, when assessed in the low-dose mode, these SEMSs obtained low scores (1.3 ± 0.6 and 2.0 ± 0.0, respectively).

## Discussion

The RF and CR of SEMSs hold substantial clinical importance. RF is essential in managing concentric strictures, while CR proves crucial for eccentric strictures. Previously, in 2009, Isayama et al. [11] examined the RF of 14 different SEMSs but did not evaluate the CR of these stents. More recently, the same group has expanded their analysis to encompass the RF of 29 different SEMSs, but once again, the CR aspect remained unexplored [12]. Our study has revealed that SEMSs with high RF do not necessarily have high CR and vice versa (Table 3). This finding suggests that certain SEMSs may be more effective for concentric strictures, while others may be better suited for eccentric strictures. For instance, the uncovered Wallflex™ Biliary RX Stent displayed low RRF and COF, yet it exhibited high CR, potentially making it more suitable for eccentric strictures. Conversely, the Zilver 635® Biliary Self-Expanding Stent demonstrated high RRF and COF but low CR, suggesting its potential suitability for concentric strictures. Both the EGIS Single Bare Biliary Stent and the E-Luminexx™ Biliary Stent showcased high RRF, COF, and CR, indicating their potential effectiveness in managing both types of strictures. In particular, the EGIS Single Bare Biliary Stent displayed notably high RRF and COF within the diameter range of 3 mm to 5 mm, implying that it may be especially suitable for tight concentric strictures.

**Table 3:** Characteristics of self-expanding metal stents based on study findings

	**Uncovered Wallflex™**	**EGIS single bare**	**Zilver 635®**	**E-Luminexx™**
**Radial resistive force**	Low	High	Moderate	High
**Chronic outward force**	Low	High	Moderate	High
**Crush resistance**	High	High	Low	Moderate
**Axial force**	High	Low	Low	High
**Conformability**	Good	Moderate	Moderate	Poor
**Surface quality**	Poor	Good	Moderate	Poor
**Foreshortening**	High	High	None	None
**Radiopacity**	Good	Poor	Good	Good

The AF and conformability of SEMSs are also of substantial clinical importance. AF refers to the SEMS’s propensity to either follow the natural curvature of a bile duct or exert force to straighten it. Conversely, conformability pertains to the SEMS’s ability to adhere closely to the duct wall at bends and curves. While previous studies have evaluated the AF of various SEMSs, the conformability of these stents has not yet been assessed [11, 12]. Our study showed that SEMSs with high AF do not necessarily exhibit high conformability, and vice versa. For instance, the uncovered Wallflex™ Biliary RX Stent exhibited high conformability but also displayed a high AF, suggesting its tendency to straighten the bile duct instead of following its natural curvature. The E-Luminexx™ Biliary Stent demonstrated a high AF and low conformability, indicating that it too would likely straighten the bile duct rather than follow its natural curvature. On the other hand, the Zilver 635® Biliary Self-Expanding Stent showed a low AF but also exhibited low conformability, implying that it may not adhere closely to the duct wall at bends and curves. Notably, among the stents evaluated, only the EGIS Single Bare Biliary Stent presented both low AF and high conformability, suggesting its potential suitability for managing torturous strictures.

Bacterial adherence and biofilm formation on biliary stents can foster an environment that leads to biliary sludge, which can eventually block the flow of bile as it accumulates [19]. It is well-recognized that the surface irregularities of the stent can promote bacterial adherence and biofilm formation, potentially increasing the likelihood of biliary sludge formation and the resultant stent occlusion [20]. Furthermore, the corrosion resistance of nitinol SEMSs, which is vital for preventing struct or wire fractures in acidic environments, is influenced by their surface condition [21]. Our study revealed distinct differences in the surface quality of the analyzed SEMSs. Notably, the EGIS Single Bare Biliary Stent demonstrated exceptional surface quality, suggesting a potential reduction in bacterial adherence and biofilm formation. Similarly, the Zilver 635® Biliary Self-Expanding Stent also exhibited favorably in this aspect. In contrast, both the uncovered Wallflex™ Biliary RX Stent and the E-Luminexx™ Biliary Stent displayed poor surface quality. This raises concerns about their susceptibility to bacterial adherence and biofilm formation.

Foreshortening and radiopacity are key determinants in facilitating the precise placement of SEMS within the bile duct. Due to their open-cell design, both the Zilver 635® Biliary Self-Expanding Stent and the E-Luminexx™ Biliary Stent exhibited no foreshortening, making placement more predictable. Furthermore, these SEMSs displayed good radiopacity, enhancing visualization during placement. On the other hand, the uncovered Wallflex™ Biliary RX Stent, despite its high radiopacity, suffers from high foreshortening, which could pose challenges in accurate placement. The EGIS Single Bare Biliary Stent, although showing lesser foreshortening than the uncovered Wallflex™ Biliary RX Stent, displayed poor radiopacity, making it less visible during placement. This high foreshortening observed in the uncovered Wallflex™ Biliary RX Stent and the EGIS Single Bare Biliary Stent is attributed to their closed-cell design. However, it is important to note that, contrary to SEMSs with open-cell designs, those with closed-cell designs usually offer the feature of being reconstrainable. In the case of the uncovered Wallflex™ Biliary RX Stent and the EGIS Single Bare Biliary Stent, both SEMSs can be reconstrained up to 80% deployment. This feature enables the repositioning of the SEMSs if their initial placement is suboptimal.

This study has several important limitations that must be considered when interpreting the results. Firstly, it is important to acknowledge that this study was conducted in an *in vitro* setting. As a result, the findings may not completely reflect the conditions present within the human body. Secondly, it is important to note that this study evaluated only four different SEMSs. However, it should be emphasized that these SEMSs are widely used for treating malignant biliary obstruction and are considered representative of common choices in clinical practice. Thirdly, statistical analysis was not conducted due to the small sample size. Lastly, each SEMS type was represented by a single sample that underwent various analyses, which could potentially affect its characteristics. However, the sequence of analyses was arranged from least to most impactful on the SEMS’s characteristics to minimize the impact on the results.

In conclusion, this study reveals considerable variation in RF, CR, AF, conformability, surface quality, foreshortening, and radiopacity among the four commonly used SEMSs for treating malignant biliary obstruction. These characteristics of SEMSs must be carefully considered during the selection process to ensure optimal therapeutic outcomes for patients.

## Data Availability

The datasets used and/or analyzed during the current study are available from the corresponding author on reasonable request.

## Abbreviations

AF: Axial force
COF: Chronic outward force
CR: Crush resistance
RF: Radial force
RRF: Radial resistive force
SEMS: Self-expanding metal stent
